# Supplementary material for: Enhanced bulk photovoltaic effect in two-dimensional ferroelectric CuInP2S6
Source: Nat Commun. 2021 Oct 8;12:5896. doi: 10.1038/s41467-021-26200-3 (PMC8501070; doi:10.1038/s41467-021-26200-3)
Supplement: Supplementary file 1 — Supplementary Information [file 41467_2021_26200_MOESM1_ESM.pdf]

Supplementary Information for "Enhanced bulk photovoltaic effect in two-dimensional ferroelectric $CuInP_2S_6$"

Yue Li[1,2,#], Jun Fu[1,2,#], Xiaoyu Mao[1,2,#], Chen Chen[1,2], Heng Liu[1,2], Ming Gong[3,4,*] and Hualing Zeng[1,2,*]

1. International Center for Quantum Design of Functional Materials (ICQD), Hefei National Laboratory for Physical Science at the Microscale, and Synergetic Innovation Center of Quantum Information and Quantum Physics, University of Science and Technology of China, Hefei, Anhui 230026, China

2. Key Laboratory of Strongly-Coupled Quantum Matter Physics, Chinese Academy of Sciences, Department of Physics, University of Science and Technology of China, Hefei, Anhui 230026, China

3. CAS Key Laboratory of Quantum Information, University of Science and Technology of China, Hefei, 230026, People's Republic of China

4. Synergetic Innovation Center of Quantum Information and Quantum Physics, University of Science and Technology of China, Hefei, Anhui 230026, China

# Contribute equally to this work

* Corresponding author

Supplementary note 1: The depolarization effect of the reading bias in CIPS bulk photovoltaics

In conventional photovoltaics, the V_{oc} is directly determined from the I-V measurement by sweeping the reading bias in a wide range. However, for 2D materials, due to the ultrathin film thickness at atomic scale, low voltage applied in the direction perpendicular to the 2D plane results in large electric field strength. For example, for films with the thickness of 1 nm, applying 1 V DC voltage leads to an electric field strength at 1×10^7 V/cm in the out-of-plane direction. This value is much larger than the coercive electric field strength of 2D ferroelectric CIPS (~7.5×10^5 V/cm as shown in Figure S2). Therefore, applying wide range reading bias in photocurrent study leads to

strong depolarization effect in CIPS. As shown in Figure S6, high bias up to -0.7 V was applied in the I-V curve measurement of device #6. The I-V curve deviates from the linearity and shrinks when the DC voltage is above -0.3 V. As a result of the depolarization effect, the nominal V_{oc} (-0.55 V) from the I-V curve should be smaller than that from the initial state of the device. By linear fit to the I-V curve, we find a much larger V_{oc} at -1.04 V (see Figure S6). To avoid this problem, when measuring the I-V curve at bright state of our devices the reading voltage is therefore limited within the range of ±0.1 V (equivalent to an electric field strength at ~10^5 V/cm). The V_{oc} is then determined through a linear fit to the I-V curve with extended DC bias range (see Fig. 2a and Fig. S6).

Supplementary note 2: The power conversion efficiency estimation of CIPS device.

We calculated the power conversion efficiency (PCE) by the formula

$$\eta = \frac{J_{sc} \times V_{oc} \times FF}{P_{in} \times \alpha}, \quad (1)$$

where J_{sc} is the short-circuit current density, V_{oc} is the open-circuit voltage in the light irradiation, P_{in} is the power of the incident light, α is the absorption coefficient of 2D CIPS, and *FF* is the fill factor, which represents the ratio between actual power and the ideal power obtained from the product $J_{sc} \times V_{oc}$. Considering the thickness of the CIPS used in our study, the ultrathin CIPS is transparent in a broad range of the light spectrum. Most intensity of the irradiation light is not absorbed by the device. To quantify the absorbance of ultrathin CIPS at 405 nm irradiation, we measure the transmission spectrum of CIPS on PDMS substrate as shown in **Fig. S15**. We find more than 97% of the light intensity transmits through the 10 nm thick CIPS. The absorption coefficient α at 405 nm is found to be lower than 3% for 10 nm CIPS. According to the data shown in **Fig. 2a** (device #2 with J_{sc} ~0.8 mA/cm^2, V_{oc} ~-1.65 V, and FF ~25 %) under on-sample light power density at 0.3 W/cm^2, the PCE of device #2 is estimated to be 3.67 %, which is more than half of the value at the S-Q limit for 2D CIPS with 2.85 eV band gap. We compare this efficiency with the conventional ferroelectrics based photovoltaic cells. For example, the PCE of 50 nm thin film ferroelectric $BaTiO_3$ is 2.1×10^{-6}. Some more data can be found in the last column of **Table I** (see the main text). Our efficiency

is appealing among all materials based on BPVE.

Supplementary figures

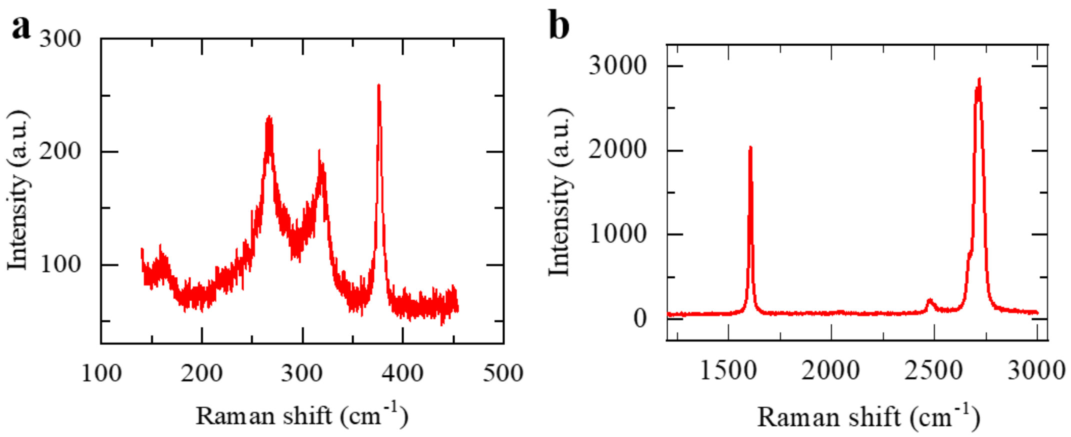

Fig. S1 Raman spectra of single crystal CIPS and bilayer graphene. (a) Raman spectrum of CIPS nanoflakes on SiO_2/Si substrate. Three dominated Raman peaks are observed. (b) Raman spectrum of the bottom graphene in device #3. Typical G mode and 2D mode peaks are observed. The intensity ratio of I_{2D} to I_G is 3/2, which indicates that the graphene used is bilayer.

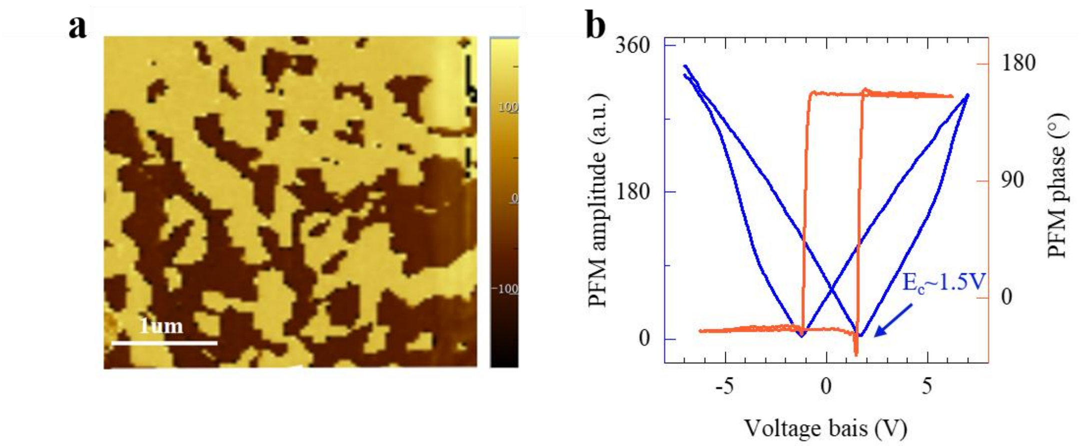

Fig. S2 PFM characterization of CIPS nanoflakes. (a) PFM phase image of the spontaneous electric polarizations in 20 nm CIPS. The scale bar is 1 μm. (b) Single-point PFM amplitude and phase hysteresis loop measurement. The coercive voltage is about 1.5 V as confirmed from the ferroelectric hysteresis loop.

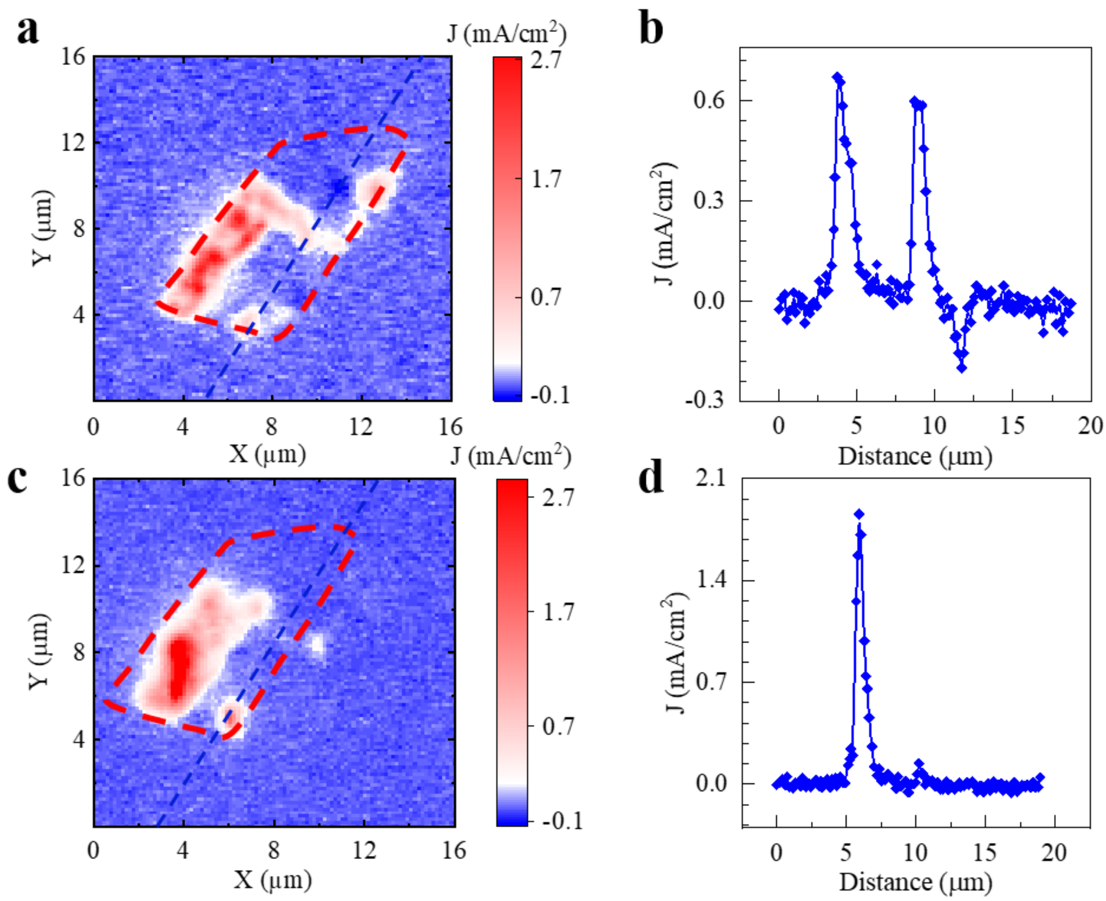

Fig. S3 Spatial confinement of the photocurrent in CIPS. (a) Zero-bias photocurrent mapping under the irradiation of 405 nm laser. (b) The spatial distribution of the photocurrent along the dotted blue line as marked in Figure S3a. Outside the overlapping area, the photocurrent is at the noise level. Inside the overlapping area, the J_{sc} is either positive or negative, which corresponds to the spontaneous downward and upward polarization in CIPS. The spatial resolution in the photocurrent mapping is 160 nm. (c) Zero-bias photocurrent mapping of the same device after 370 K annealing. Upon annealing, the spontaneous electric polarizations in CIPS are renovated. Correspondingly, the pattern of the J_{sc} mapping changes if compared with Figure S3a. (d) The spatial distribution of the photocurrent along the dotted blue line as marked in Figure S3c. Due to the thermal-induced ferroelectric polarization redistribution the negative photocurrent disappears.

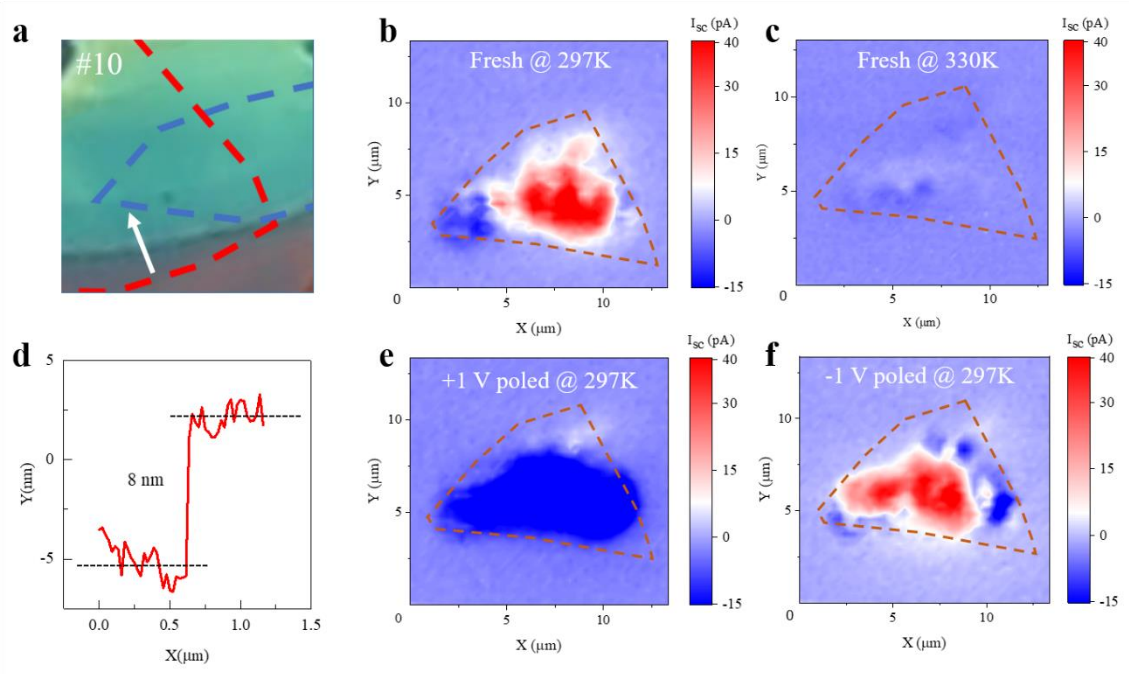

Fig. S4 Dependence of the zero-bias photocurrent mapping on poling voltage and temperature. (a) The optical image of device #10. (b)&(c) The zero-bias photocurrent mapping on fresh device under the irradiation of 405 nm laser at 297K and 330K. Above T_c, negligible I_{sc} is observed. (d) The AFM measurement of the CIPS in device #10 as indicated by the white arrow in Figure S4a. The thickness of the CIPS used in device #10 is 8 nm. (e) & (f) The zero-bias photocurrent mapping under the irradiation of 405 nm laser after +1 V and -1 V voltage polarized. Clear sign change of the I_{sc} is observed, indicating the switchable BPVE in CIPS.

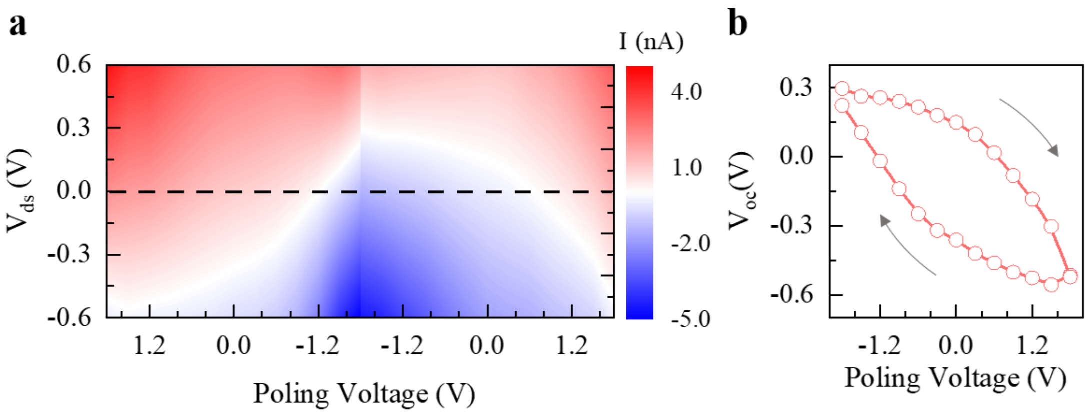

Fig. S5 Extended electrically switchable BPVE in CIPS. (a) The extended I-V curve in device #3 with a linear fit. (b) The V_{oc} as a function of the poling voltage.

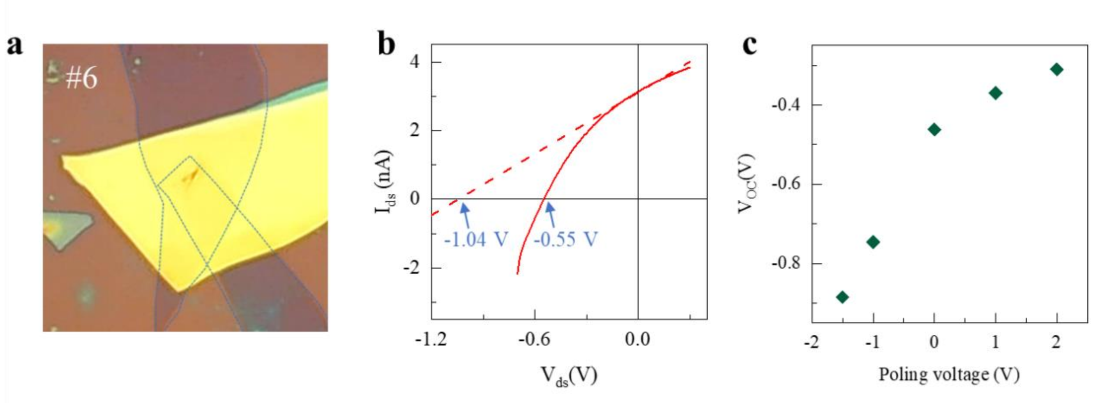

Fig. S6 Depolarization effect of the reading bias in BPVE measurement. (a) The optical image of device #6. The blue dotted lines indicate the area of the top and bottom graphene electrodes. (b) The photovoltaic characteristic measured in a wide range reading voltage from -0.7 V to 0.3 V. The I-V curve deviates from linear when the reading voltage is larger than -0.3 V. By linear fit (red dashed line) to the I-V curve, we get a larger V_{oc} at -1.04 V (c) The V_{oc} dependence on poling voltage.

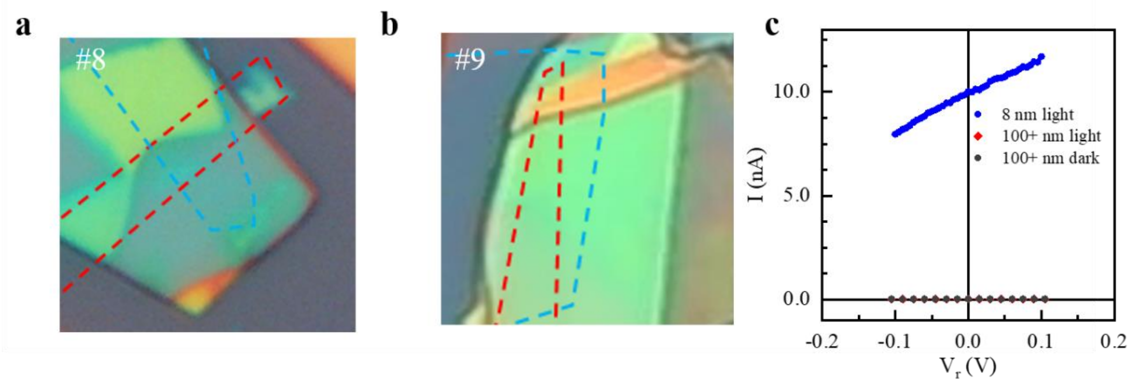

Fig. S7 Photovoltaic effect study on CIPS with film thickness above 100 nm. (a) & (b) The optical images of device #8 & device #9. The blue and red dotted shapes indicate the bottom and top graphene electrodes respectively. The thickness of the CIPS film in device #8 and device #9 are 120 nm and 230 nm respectively, which are confirmed by AFM (Figure S9h & S9i). (c) The photovoltaic characteristic of 8 nm and above 100 nm CIPS under the irradiation of 405 nm laser at same power density, the CIPS above 100 nm shows photocurrent at the order of noise level and coincides with the dark current.

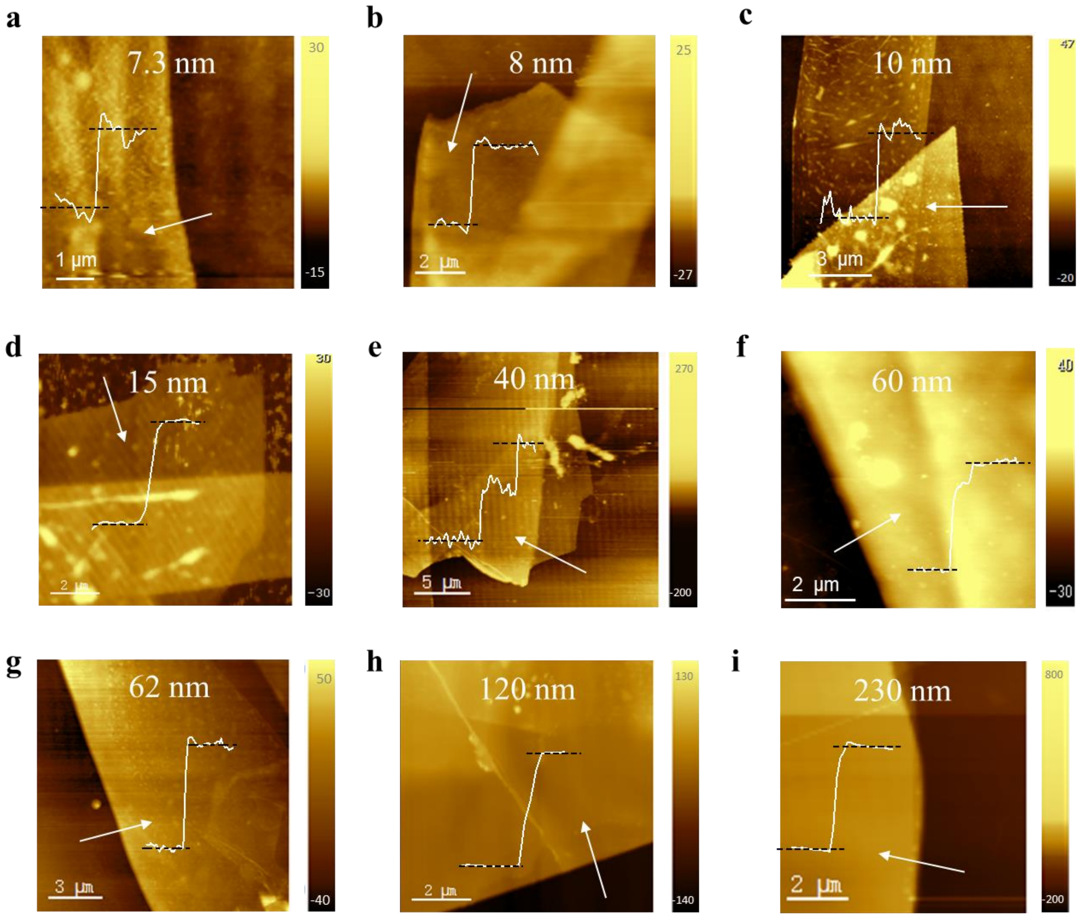

Fig. S8 Thickness identification and AFM topography for different CIPS samples. (a) – (i) AFM image with height profile of the CIPS samples used in Fig. 5b with film thickness ranging from 7 nm to 230 nm. The scale bar is about several µm subject to the different sample sizes.

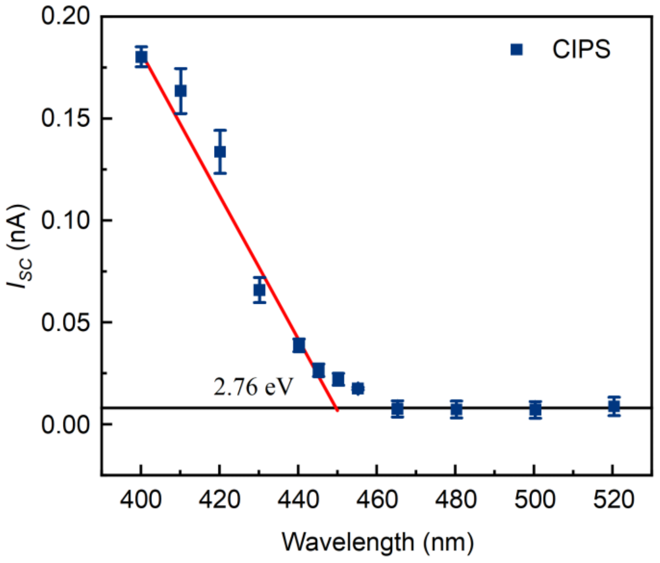

Fig. S9 Wavelength spectra of the BPVE in CIPS. The zero-bias photocurrent with each wavelength laser irradiation is plotted as a function of laser wavelength.

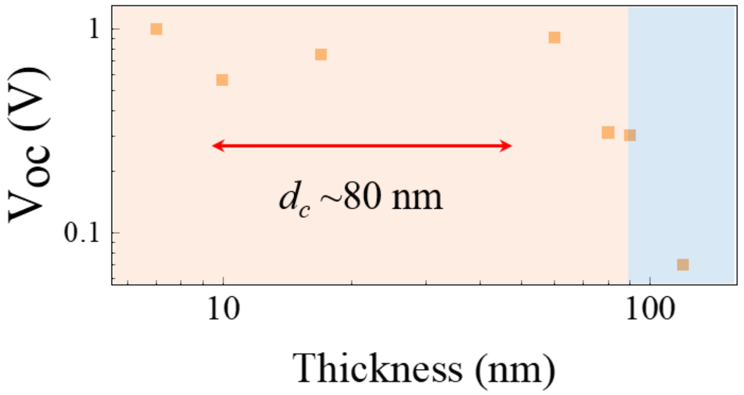

Fig. S10 Open-circuit photovoltage of CIPS devices with various thicknesses. When the thickness is less than the diffusion length, V_{oc} is independent of the thickness; however, when the thickness exceeds the diffusion length, V_{oc} quickly drops to zero.

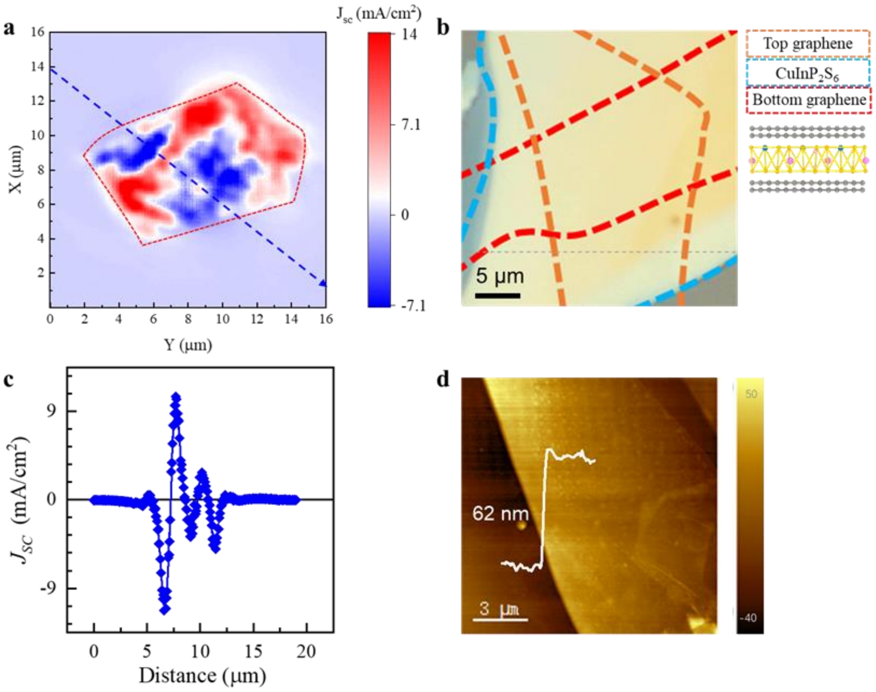

Fig. S11 High resolution zero-bias photocurrent mapping. (a) Zero-bias photocurrent mapping with a higher resolution. (b)Photograph of the CIPS device, the yellow, blue and red dotted line represent the top graphene, CIPS and bottom graphene respectively. (c) The photocurrent along the dotted line. (d) The AFM topography of CIPS used in the device, inset is the AFM line profile.

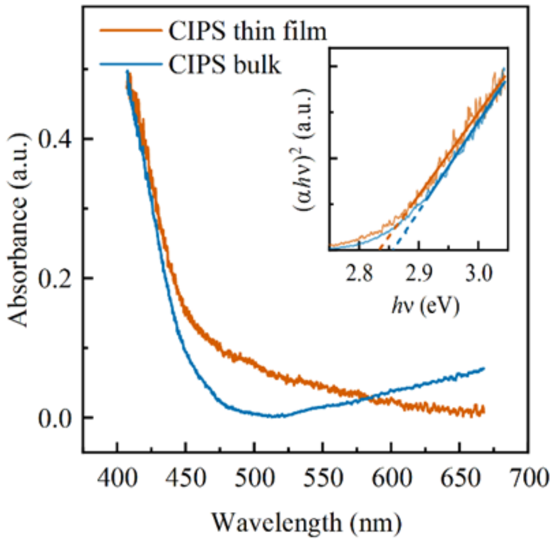

Fig. S12 Absorption Spectrum of CIPS on transparent PDMS substrate. The linear fitting near 2.8 – 2.9 eV yields the energy gap.

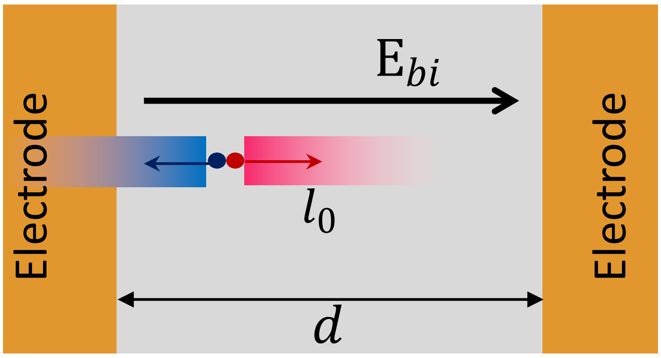

Fig. S13 Illustration of diffusion process in carriers been created by irradiation. The electron and hole will be driven The created electron and hole can reach the two electrodes when the distance is less than $2l_0$, where l_0 is the carrier diffusion length. Here we assume the electron and hole have the same diffusion length. When $d > 2l_0$, the created electron and hole cannot reach the electrodes simultaneously, yielding a sudden drops of photocurrent.

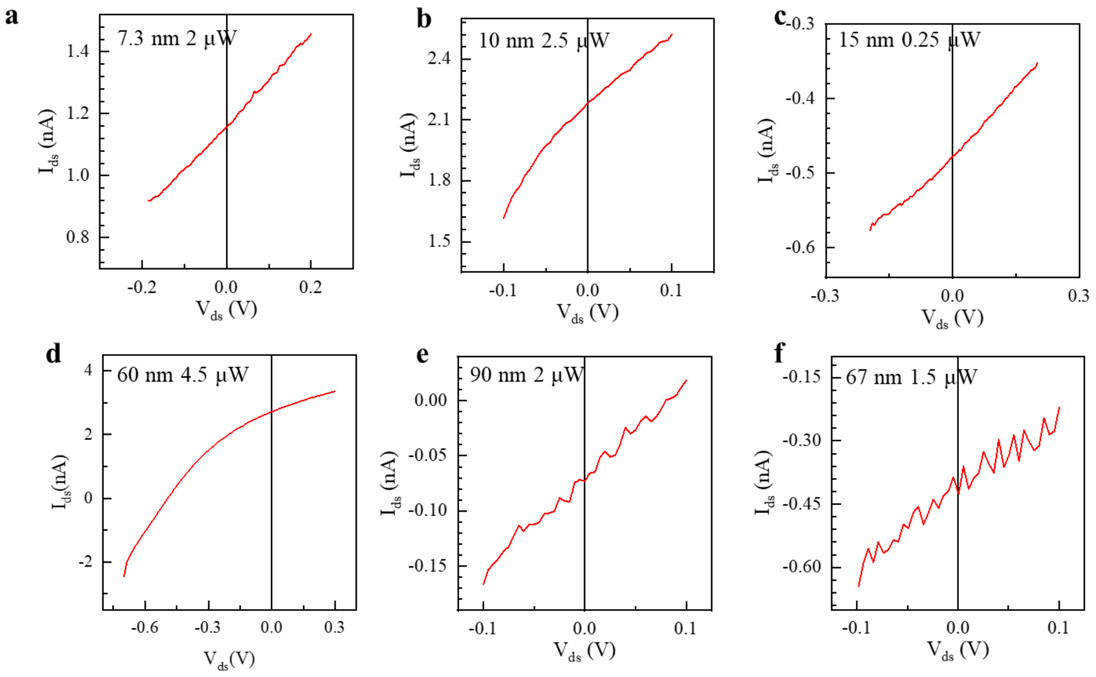

Fig. S14 Photovoltaic transport character of devices in Fig. 5b for different thicknesses (in the unit of nm) and different excitation power (in the unit of μW). The intercept photocurrent at zero bias (for example in (a), $I_{sc} = I_{ds}(V_{ds} = 0)$ is 1.2 nA) divided by the excitation laser spot size yields the photo response density used in the main text.

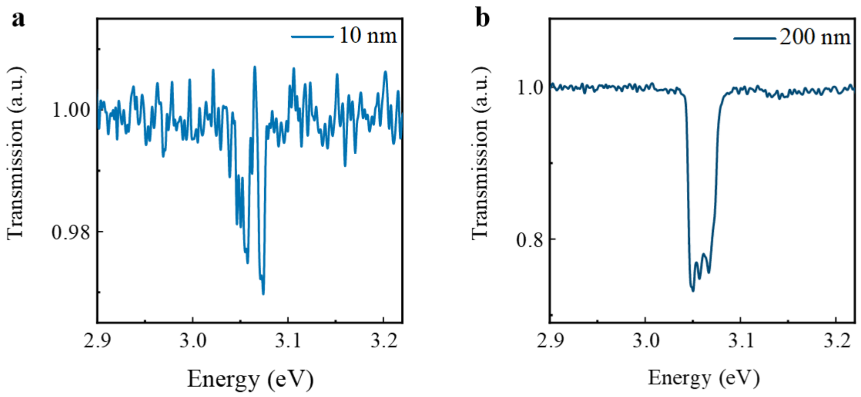

Fig. S15 Transmission spectra of 10 nm (a) and >200 nm (b) CIPS thin films measured at 405 nm. In our experiment, the 405 nm laser is adopted as the irradiation source. The transmission (T) is defined as $\mathrm{T} = I_{\mathrm{CIPS}}/I_{\mathrm{sub}}$, where I_{CIPS} and I_{sub} are the transmitted light intensity of CIPS on PMDS and bare PDMS substrate. We estimate the transmission coefficient for 10 nm CIPS to be about 97%, which is used in the main text for the estimation of PCE.
